# Predicting the Evolution of Sex on Complex Fitness Landscapes

**DOI:** 10.1371/journal.pcbi.1000510

**Published:** 2009-09-18

**Authors:** Dusan Misevic, Roger D. Kouyos, Sebastian Bonhoeffer

**Affiliations:** Institute of Integrative Biology, ETH Zürich, Zürich, Switzerland; University of Washington, United States of America

## Abstract

Most population genetic theories on the evolution of sex or recombination are based on fairly restrictive assumptions about the nature of the underlying fitness landscapes. Here we use computer simulations to study the evolution of sex on fitness landscapes with different degrees of complexity and epistasis. We evaluate predictors of the evolution of sex, which are derived from the conditions established in the population genetic literature for the evolution of sex on simpler fitness landscapes. These predictors are based on quantities such as the variance of Hamming distance, mean fitness, additive genetic variance, and epistasis. We show that for complex fitness landscapes all the predictors generally perform poorly. Interestingly, while the simplest predictor, *ΔVar_HD_*, also suffers from a lack of accuracy, it turns out to be the most robust across different types of fitness landscapes. *ΔVar_HD_* is based on the change in Hamming distance variance induced by recombination and thus does not require individual fitness measurements. The presence of loci that are not under selection can, however, severely diminish predictor accuracy. Our study thus highlights the difficulty of establishing reliable criteria for the evolution of sex on complex fitness landscapes and illustrates the challenge for both theoretical and experimental research on the origin and maintenance of sexual reproduction.

## Author Summary

One of the biggest open questions in evolutionary biology is why sexual reproduction is so common despite its manifold costs. Many hypotheses have been proposed that can potentially explain the emergence and maintenance of sexual reproduction in nature, and currently the biggest challenge in the field is assessing their plausibility. Theoretical work has identified the conditions under which sexual reproduction is expected. However, these conditions were typically derived, making strongly simplifying assumptions about the relationship between organisms' genotype and fitness, known as the fitness landscape. Building onto previous theoretical work, we here propose different population properties that can be used to predict when sex will be beneficial. We then use simulations across a range of simple and complex fitness landscapes to test if such predictors generate accurate predictions of evolutionary outcomes. We find that one of the simplest predictors, related to variation of genetic distance between sequences, is also the most accurate one across our simulations. However, stochastic effects occurring in small populations compromise the accuracy of all predictors. Our study both illustrates the limitations of various predictors and suggests directions in which to search for new, experimentally attainable predictors.

## Introduction

Sexual reproduction is widespread among multi-cellular organisms [1]. However, the ubiquity of sex in the natural world is in stark contrast to its perceived costs, such as the recombination load [2] or the two-fold cost of producing males [3],[4]. Given these disadvantages it is puzzling that sexual reproduction has evolved and is maintained so commonly in nature. The “paradox of sex” has been one of the central questions in evolutionary biology and a large number of theories have been proposed to explain the evolution and maintenance of sexual reproduction [5]. Currently, the most prominent theories include (i) the Hill-Robertson effect [6]–[8], (ii) Muller's ratchet [9], (iii) the Red Queen hypothesis [10],[11], and (iv) the Mutational Deterministic hypothesis [12],[13]. While originally described in various different ways, the underlying benefit of sex can always be related to the role of recombination in breaking up detrimental statistical associations between alleles at different loci in the genome. What fundamentally differentiates the theories is the proposed cause of these statistical associations, assigned to either the interactions between drift and selection (Fisher-Muller effect, Muller's ratchet, and Hill-Robertson effect) or gene interactions and epistatic effects (Red Queen hypothesis and Mutational Deterministic hypothesis).

The present list of hypotheses is certainly not exhaustive, with new ones continuously being proposed, complementing or replacing the existing ones [14]. However, it is not new hypotheses that are most needed, but the real-world evidence that allows us to distinguish between them. The major question that still remains is whether the assumptions and requirements of different theories are fulfilled in the natural world. Accordingly, there has been considerable effort to experimentally test these assumptions, mainly for the epitasis-based theories (reviewed in [15]–[17]). However, an even more basic and crucial problem underlies all work on evolution of sex: how does one choose, measure, and interpret appropriate population properties that relate to different theories [17]–[19]. The difficulty stems from the often large divide between the theoretical and experimental research: theories are frequently formulated as mathematical models and rely on simplistic fitness landscapes or small genome size (e.g. two locus, two allele models) [13], [20]–[25]. As a result, it may be unclear how a property established based on these simplified assumptions relates to actual properties of natural populations.

In this study we attempt to bridge the gap between the theoretical and experimental work and to identify which population measures are predictive of the evolution of sexual reproduction by simulating the evolution of both sexual and asexual populations on fitness landscapes with different degrees of complexity and epistasis. The measures we use are the change of mean fitness, of additive genetic variance, or of variance in Hamming distance as well as four epistasis-based measures, physiological, population, mean pairwise, and weighted mean pairwise epistasis. While this certainly is not an exhaustive list, we took care to include major quantities previously considered in theoretical and experimental literature (e.g. [26]–[28]). With some exceptions [29]–[32], earlier work generally focused on the smooth, single peaked landscapes, while here we also use random landscapes and NK landscapes (random landscapes with tunable ruggedness). Some studies of more complex rugged landscapes tested whether they would select for sex but have not found a simple and unique answer, even in models with only two-dimensional epistasis [33],[34]. A recent paper, which uniquely combines experimental and theoretical approaches and simulates evolution of sex on empirical landscapes, also finds that landscape properties greatly affect the outcome of evolution, sometimes selecting for but more often against sex [35]. However, what specifically distinguishes our study is the goal of not only determining when sex evolves but also of quantifying our ability to detect and predict such outcome in scenarios where we know how the evolution proceeds.

Whether the more complex landscapes we are using here are indeed also more biologically realistic is open to debate as currently little is known about the shape and the properties of real fitness landscapes (for an exception see for example [35],[36]). Our goal is to move the research focus away from the simple landscapes mostly investigated so far to landscapes with various higher degrees of complexity and epistasis, and to probe our general understanding of the evolution of sexual reproduction on more complex fitness landscapes.

Notably, we find that some of the measures routinely used in the evolution of sex literature perform poorly at predicting whether sex evolves on complex landscapes. Moreover, we find that genetic neutrality lowers the predictive power of those measures that are typically robust across different landscapes types, but not of those measures that perform well only on simple landscapes. The difficulty of predicting sex even under the ideal conditions of computer simulations, where in principle any detail of a population can be measured with perfect accuracy, may be somewhat sobering for experimentalists working on the evolution of sex. We hope, however, that this study will evoke interest among theoreticians to tackle the challenge and develop more reliable predictors of sex that experimentalists can use to study the evolution of sex in natural populations.

## Results

### Quality of predictors

We investigated the evolution of sex in simulations on three types of fitness landscapes with varying complexity (smooth, random and NK landscapes) and used seven population genetic quantities (*ΔVar_HD_*, *ΔVar_add_*, *ΔMean_fit_*, *E_phys_*, *E_pop_*, *E_MP_*, and *E_WP_*, Table 1) as predictors of change in frequency of the recombination allele (see Methods for more details). We calculated predictor accuracy (the sum of true positives and true negatives divided by the total number of tests) and used it to assess their quality on 110 smooth landscapes with varying selection coefficients and epistasis, 100 random landscapes, and 100 NK landscapes each for *K = 0,…,5*. All landscapes are based on 6 biallelic loci and they were generated such that an equal number of landscapes of each type select for versus against sex in deterministic simulations with infinite population size. Hence, random prediction by coin flipping is expected to have an accuracy of 0.5.

**Table 1:** Overview of predictors of the evolution of sex.

Predictor	Brief description	Sex evolves if
*ΔVar_HD_*	Difference in variance of Hamming distance to the consensus sequence after full and before recombination	*ΔVar_HD_*>0
*ΔVar_add_*	Difference in additive genetic variance after full and before recombination	*ΔVar_add_*>0
*ΔMean_fit_*	Difference in mean fitness after full and before recombination	*ΔMean_fit_*>0
*E_phys_*	Physiological epistasis characterizing fitness landscape	*E_phys_<*0
*E_pop_*	Population epistasis characterizing epistatic interactions observed in population	*E_pop_<*0
*E_MP_*	Mean pairwise epistasis (from [28])	*E_MP_<0*
*E_WP_*	Weighted mean pairwise epistasis (from [28])	*E_WP_<0*

**Figure 1:**
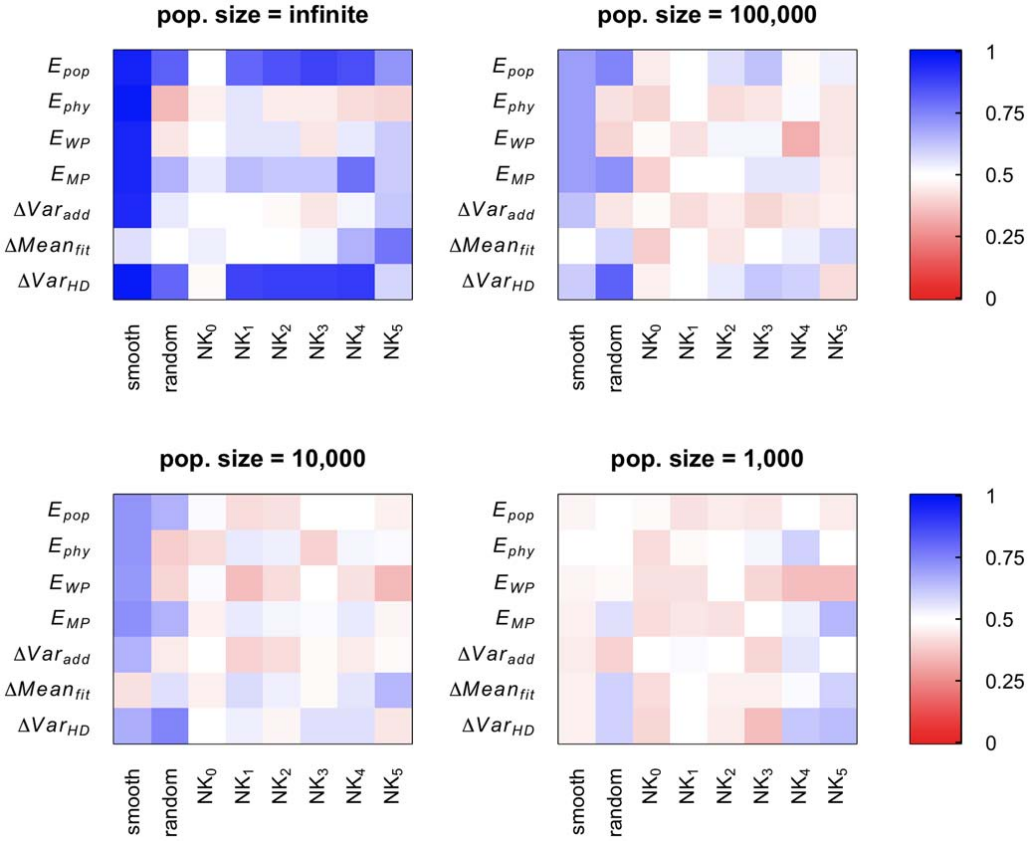
Predictor accuracy for different landscape types. Panels correspond to simulations with different population size. Predictors with absolute values smaller than 10^−15^ were considered numerical artifacts and were instead assigned values of −1 or 1 at random. This was done in particular for NK_0_ landscapes where epistasis is always 0 and thus selection for or against sex is absent in infinite populations. Such substitution is appropriate, because all predictors rely on sign and not magnitude in predicting the outcome of the competition phase.

Figure 1 shows the accuracy of the predictors for the different landscape types. Increasing levels of blue indicate greater accuracy of prediction. For the simulations with infinite population size (deterministic simulations) we ran a single competition between sexual and asexual populations to assess whether sex was selected for. For simulations with finite population size (stochastic simulations), we ran 100 simulations of the competition phase and assessed whether the predictor accurately predicts the evolution of sex in the majority of these simulations. Focusing on the top left panel we find that for deterministic simulations most predictors are only highly accurate in predicting evolutionary outcomes for the smooth landscapes. The exception is the poor performance of *ΔMean_fit_*, which is not surprising, as theory has shown that for populations in mutation-selection balance *ΔMean_fit_* is typically negative [2]. According to our use of *ΔMean_fit_* as a predictor, it always predicts no selection for sex when negative and thus is correct in 50% of cases, due to the way the landscapes were constructed. For the NK_0_ landscapes, all predictors perform poorly, because such NK landscapes have no epistasis by definition (see Methods). For infinite population size, theory has established that in absence of epistasis there is no selection for or against sex. Indeed, in our simulations the increase or decrease in the frequency of sexual individuals is generally so small (of order 10^−15^ and smaller) that any change in frequency can be attributed to issues of numerical precision. Generally, the accuracy of most predictors is much weaker for complex landscapes (NK and random landscapes) than for the simpler, smooth landscapes. The predictors that have highest accuracy across different landscape types are *ΔVar_HD_* and *E_pop_*.

### Combining predictors

**Figure 2:**
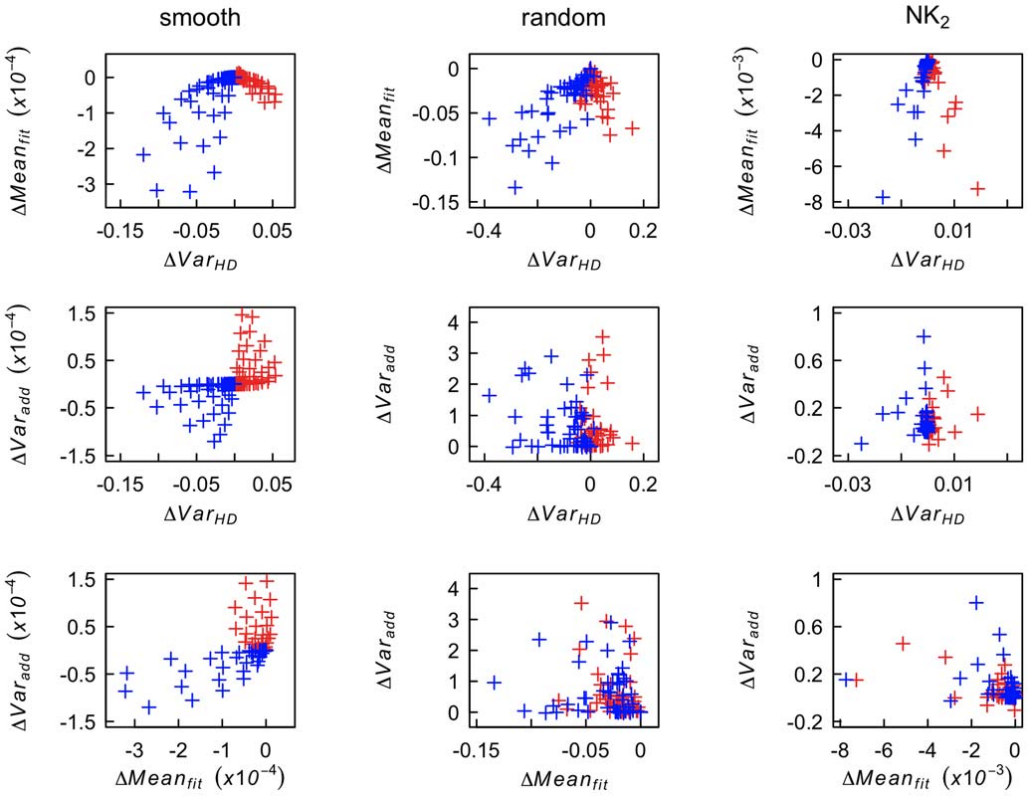
Correlations between predictors on different landscapes. We highlight the relationships among *ΔVar_HD_*, *ΔVar_add_*, and *ΔMean_fit_* on smooth, random, and NK_2_ landscapes, in simulations with infinite population size. Each cross mark (+) represents a predictor value for a single simulation. Red (blue) crosses indicate simulations in which the frequency of sexually reproducing individuals increased (decreased) in the competition phase. For clarity of presentation, up to 5 outlier points were eliminated from random and NK_2_ landscapes. These outliers in predictor values are typically characteristic of a small number of populations that did not reach the equilibrium genotype frequencies by the end of the burn-in phase.

To test whether combinations of the predictors could increase the accuracy of prediction of the evolution of sex we plot for each landscape the value of the predictors *ΔVar_HD_*, *ΔVar_add_* and *ΔMean_fit_* against each other and color code whether the number of sexual individuals increased (red) or decreased (blue) during deterministic competition phase (see Figure 2). If the blue and red points are best separated by a vertical or a horizontal line, then we conclude that little can be gained by combining two predictors. If, however, the points can be separated by a different linear (or more complex) function of the two predictors, then combining these predictors would indeed lead to an improved prediction. Figure 2 shows the corresponding plots for the smooth, the random, and the NK_2_ landscapes. For the smooth landscapes the criterion *ΔVar_HD_>0* or *ΔVar_add_>0* are both equally good in separating cases where sex evolved from those where it did not. As already shown in Figure 1, *ΔVar_HD_* is generally a more reliable predictor of the evolution of sex than *ΔVar_add_* in the more complex random or NK landscapes. Epistasis-based theories suggest that the selection for sex is related to a detrimental short-term effect (reduction in mean fitness) and a possibly beneficial long-term effect (increase in additive genetic variance) [28]. The plots of *ΔVar_add_* against *ΔMean_fit_*, however, do not indicate that combining them would allow a more reliable prediction of the evolution of sex. Generally, the plots show that blue and red points either tend to overlap (in the more complex landscapes) or can be well separated using horizontal or vertical lines (in the smooth landscapes) such that combining predictors will not allow to substantially increase the accuracy of prediction. This is also the case for all other landscapes and all other pairwise combinations of predictors (data not shown). It is possible that some of the effect described in [28] and expected here are too small to be detected with the level of replication in our study. However, as the level of replication used in this computational study goes way beyond what can be realistically achieved in experimental settings we expect that these effects would also not be detected in experimental studies.

We also used a linear and quadratic discriminant analysis to construct functions to predict the outcome of competitions between the two modes of reproduction. For these purposes, half of the data set was used for training and the other half for testing of the discriminant functions, and the procedure was repeated separately for each of the three population sizes (1,000, 10,000, and 100,000) and the deterministic case. In no case did these methods improve the accuracy of predictions (data not shown). While there certainly are other, potentially more sophisticated techniques that could be used here, our analysis indicates that there may not be much additional information in our metrics that could be extracted and used to increase the accuracy of the predictions.

### Effects of population size

**Figure 3:**
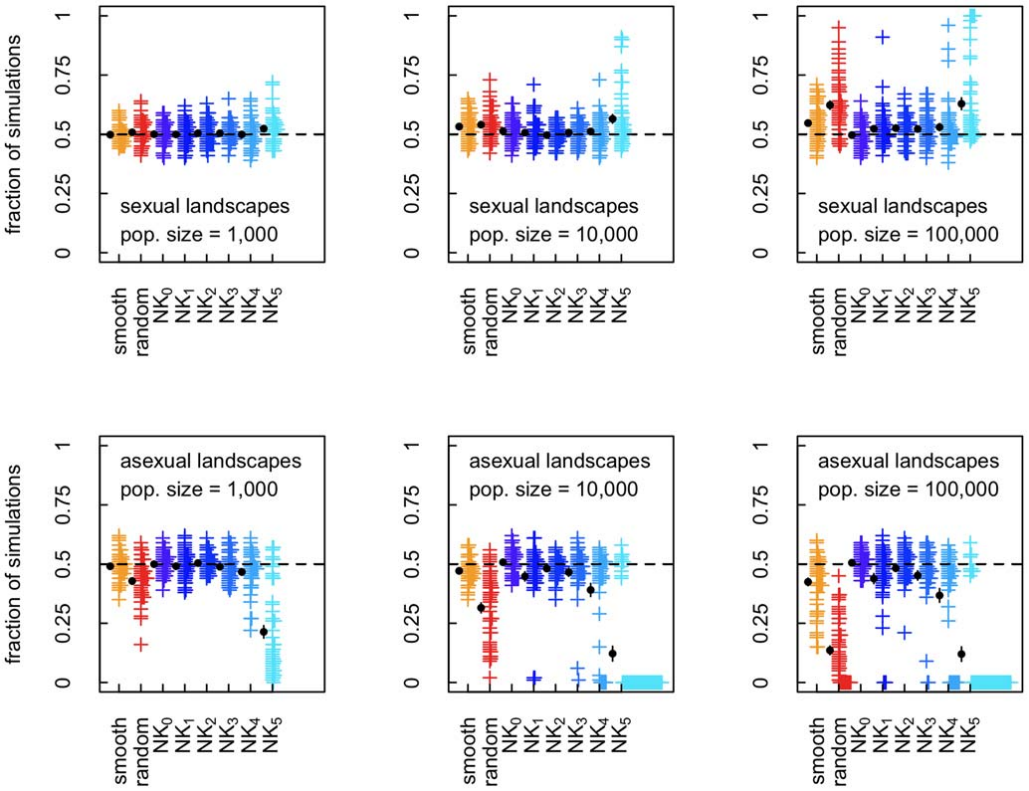
Fraction of simulations in which sex evolved on different types of landscapes for finite population sizes. For each population size and each landscape we performed 100 simulations. Each cross mark (+) represents the results for one particular landscape, offset horizontally for visibility, calculated as the fraction of those 100 simulations in which sex increased in frequency for a given landscape. The top and bottom row of panels corresponds to landscapes that select for sex versus asex in deterministic simulations. For each landscape type, mean and one standard error of the mean are shown on the left side of the cross marks. In some cases, the standard error is too small to be visible on the plot and is covered by the circle marking the mean value.

All predictors performed much worse for simulations with finite population size (Figure 1), most likely because the selection coefficient for sex is weak [19],[20]. To further examine the effect of finite population size on the evolution of sex on different landscape types we analyzed 100 independent simulations of the competition phase starting from the genotype frequencies obtained from the burn-in phase on each landscape. Figure 3 shows the fraction of cases in which the frequency of sexual individuals increased for three population sizes (1,000, 10,000, and 100,000), plotted separately for those landscapes in which frequency of the recombination modifier increased or decreased in deterministic simulations. For almost all landscapes the fraction of cases in which sex evolves is close to 50%, indicating that selection for sexual reproduction is indeed extremely weak, and can thus easily be overwhelmed by stochastic effects (in contrast to simulations with infinite populations where selection coefficients of any size will always produce a consistent observable effect). As a consequence, even for relatively large population sizes the outcome of the competition between sexual and asexual populations is largely determined by drift. Such weak selection may in part due to the small number of loci used for these simulations and stochastic simulations with larger genomes have indeed been shown to result in stronger selection for or against sex [37],[38]. However, accurate deterministic simulations are computationally not feasible for large genome sizes, because of the need to account for the frequency of all possible genotypes in deterministic simulations (see Supporting Information (Text S1) for more details).

According to the Hill-Robertson effect (HRE) [8],[21] selection for recombination or sex may be stronger in populations of limited size, because in such populations the interplay between drift and selection can generate negative linkage disequilibria, which in turn select for increased sexual reproduction. The strength of HRE vanishes for very small populations and for populations of infinite size [21]. In an intermediate range of population sizes, the HRE increases with increasing number of loci (as does the range of population sizes in which the effect can be observed) [38] and for large genome size it can be strong enough to override the effect of weak epistasis [37]. In our simulations, however, HRE is weak, as is evidenced by the fact that, in the NK_0_ landscape, which by definition have no epistasis, the fraction of runs in which sex evolves is only very marginally above 50% (Figure 3).

Our results indicate that for finite population size the predictors generally perform poorly. Of course this does not imply that they could not be better than a simple coin toss. However, the results suggest that these predictors will likely be of limited use, as any experiment will have difficulties to reach even the replicate number that we have used to generate Figure 1.

### Effects of neutrality

We also examined additional fitness landscapes, characterized by increased neutrality (for full details and figures see Text S1). We found that the allelic diversity at neutral loci both decreases the accuracy and generates a systematic bias in the previously best performing predictors, *E_pop_* and *ΔVar_HD_*. In contrast, other predictors investigated here, *ΔVar_add_*, *ΔMean_fit_*, *E_phys_*, *E_MP_*, and *E_WP_* are not affected by including neutral loci, but still have poor accuracy of prediction on more complex fitness landscapes.

## Discussion

Our computer simulations highlight the difficulties in predicting whether the frequency of sexual individuals will increase in populations evolving on complex fitness landscapes. The predictors of the evolution of sex used here are derived from previous studies on simpler landscapes and are based on standard population genetic measures such as variance of Hamming distance, mean fitness, additive genetic variance, physiological or population epistasis, mean pairwise epistasis and weighted mean pairwise epistasis. Not surprisingly, all predictors are highly accurate on the simplest landscape type, the smooth landscapes, in which log fitness is a monotonic, weakly curved function of the Hamming distance to the fittest sequence. Interestingly, the simplest measure, *ΔVar_HD_*, which is based on the change of Hamming distance after versus before recombination, turns out to be among the most robust predictors of the evolution of sex across the range of fitness landscapes tested here (Figure 1). Notably, *ΔVar_HD_* requires no fitness data, but only genetic information, and should thus be easier to obtain experimentally, at least when compared to the measures that require both information on the mutations present and on their fitness effects. Intuitively, *ΔVar_HD_* measures whether recombination has the effect of spreading out a population or condensing it over the space of all possible genotypes. Spreading out the population over genotype space (i.e. increasing genetic variation) may increase phenotypic variation, which in turn leads to more efficient selection on fitness affecting loci and eventually to selection for sexual reproduction. Another measure, population epistasis, *E_pop_*, turns out to be an equally robust predictor of the evolution of sex. *E_pop_* may be more convenient than *E_phys_* in experimental studies because there is no need to generate a large number of mutants for the analysis. However, neither of these two predictors manages to attain a high degree of accuracy on complex landscapes under stochastic conditions. Using combinations of predictors also does not appear to increase the overall ability to predict evolutionary outcome in our simulations (Figure 2). Our results are in general agreement with previous work on the evolution of sex on rugged and complex landscapes [32]–[35]. For example, we had to generate many landscapes before finding 50 of each type on which sex evolves, with sometimes less than 1 in 10 landscapes promoting sex under the deterministic scenario (data not shown.)

Finding such poor performance of all the predictors is a somewhat sobering result. A possible criticism of our approach is that we have focused in our simulations on small genomes in mutation selection balance. In such a situation the selection for sex is particularly weak and hence likely to be overwhelmed by stochastic effects. An alternative scenario for which effects could be stronger is that of populations in which a substantial fraction of beneficial mutations have not yet gone to fixation [21]. Preliminary simulations of this alternative scenario suggest that, under a narrow range of parameters (low mutation rate, single mal-adapted founding genotype, large population size), sex indeed evolves more frequently than in simulations starting from mutation selection balance (data not shown). Generally, however, the quality of the predictors does not substantially increase. More work is needed to characterize and fully examine predictors in adapting populations, highlighting these scenarios as interesting future directions, but outside of the scope of the present study.

Our goal here was not to address all of the different theories on evolution of sex and our simulations are certainly not well suited for investigating the Red Queen hypothesis ([10] and for a recent review see [39]) which is based on fluctuating selection or the Fisher-Muller hypothesis [6],[7],[21] which is based on the effect of beneficial mutations. To do so properly would require an entirely new setup, including for example, changing fitness landscapes and/or presence of parasitic individuals in case of the Red Queen hypothesis and the continuous presence of novel beneficial mutations in case of the Fisher-Muller hypothesis. Instead our study focuses on those hypotheses for the evolution of sex/recombination such as the Mutational Deterministic hypothesis [12],[13] or the Hill-Robertson effect [8],[37] that work at mutation-selection balance.

### Conclusion

The central message of our study is that the prediction of the evolution of sex is difficult for complex fitness landscapes, even in the idealized world of computer simulations where in principle one can measure any detail of a given population and fitness landscape. Here we put the emphasis on predictors that are experimentally measurable and are based on conditions for the evolution of sex established in the population genetic literature using simple fitness landscapes. We have however included *E_MP_* and *E_WP_*, two predictors which would be more difficult to measure experimentally, but are based on the most fundamental and general theoretical treatment of the evolution of sex [28]. Of course, while our choice of predictors, landscapes and selection regimes is comprehensive, we are aware that it can never be exhaustive or complete – there will always be other options to try out and test. Future work will have to focus on identifying more reliable predictors of the evolution of sex that can be used in conjunction with experimental data. Additionally, a better characterization of properties of natural fitness landscapes is badly needed to improve our understanding of the forces selecting for the evolution of sex. As it stands, *ΔVar_HD_*, our best candidate for a predictor of the evolution of sex, has nevertheless important shortcomings. In particular, it never reaches high levels of accuracy on many of the landscapes. Still, *ΔVar_HD_* at least suggests a potential direction for future research: a focus on predictors that would take advantage of the rapidly increasing number of fully or partially sequenced genomes and allow us to determine the advantage of sex in large numbers of taxa, bringing us closer to fully understanding the evolution of sex.

## Materials and Methods

### Fitness landscape types

#### Smooth landscapes

The log fitness *w_i_* of a genotype *i* is given by 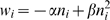, where *n_i_* is the Hamming distance from the fittest genotype (i.e. the number of loci by which genotype *i* differs from the fittest genotype). The parameters *α* and *β* determine the slope and curvature of the logarithm of the fitness function. A positive (negative) value of *β* corresponds to positive (negative) epistasis. The parameter *α* is confined to positive values. The maximal Hamming distance is given by the total number of loci, *N* = 6. To ensure that fitness decreases monotonically with increasing Hamming distance we confined *β* to values between −*α/(2 N)* and *α/(2 N)*. We generated a set of 110 smooth landscapes by choosing 10 values of *α* equally spaced between 0.1 and 0.001 on log scale (*α* = 10^−(1+2(*i*−1)/9)^, for *i* = 1,…,10) and 11 equally spaced values of *β* between *−α/(2 N)* and *α/(2 N)*. Thus we have 50 pairs of smooth landscapes with corresponding levels of positive and negative epistasis and 10 landscapes with no epistasis. For populations of infinite size, landscapes with positive epistasis are predicted to select against sex and landscapes with negative epistasis select for sex [28], which our simulations confirmed.

#### Random landscapes

For random landscapes the fitness values of all genotypes are random numbers independently drawn from uniform distribution on [0, 1] interval. Due to such construction, random landscapes are maximally epistatic in the sense that the fitness is not determined by contributions of individual loci, but depends entirely on the combination of the alleles at all loci. However, in contrast to the smooth landscapes, we do not directly set the particular epistatic values for the random landscapes. Another way of characterizing random landscapes would be to point to the complete lack of heritability – the fitness of a mutant progeny is entirely independent of the parental fitness. We generated 100 random landscapes with 50 each selecting for or against sex, based on whether sexual individuals outcompete asexual ones in simulations with infinite population size.

#### NK landscapes

NK landscapes are random landscapes of tunable ruggedness, first introduced by and described in detail by Kauffman [40]. In brief, *N* denotes the total number of loci that define the landscape and *K* denotes the number of loci with which each locus interacts. The fitness of a genotype is determined as follows: For each locus we randomly choose a set of *K* other loci (*K≤N−1*). Then, for each locus, we generate a look-up table that assigns a random number drawn uniformly between 0 and 1 to each of the 2*^K^* ^+1^ possible combinations of alleles at the *K* interacting loci and the focal locus. To obtain the fitness of a given genotype we multiply the fitness contributions from each locus, determined form the corresponding look-up tables, and scale the result so that the fittest genotype has fitness one. Landscapes with *K* = 0 (NK_0_), are single peaked and free of epistasis. In contrast, the landscapes with *K = N−1* (NK_N−1_) are highly epistatic, multi-peaked random landscapes, where the fitness of each genotype is the product of *N* uniformly distributed random numbers. (Note that commonly in NK landscapes the fitness is defined as the sum rather than the product of the contributions of all loci. We deviate from the common definition to ensure that NK_0_ landscapes have no epistasis on a multiplicative scale). For each value of *K* we generated 100 landscapes, 50 selecting for and 50 against sex, based on whether sexual individuals outcompete asexual ones in populations of infinite size.

### Simulations

All simulations of the evolution of a haploid population on a given fitness landscape are divided into a “burn-in” and a “competition” phase. In the burn-in phase an asexually reproducing population is allowed to equilibrate on the landscape starting from random initial genotype frequencies. In the competition phase we determine whether the frequency of an allele coding for increased recombination increases in the population.

The burn-in phase consists of repeated cycles of mutation and selection. Genotype frequencies after selection are given by the product of their frequency and relative fitness before selection. In all simulations mutations occur independently at each locus with a mutation rate *μ* = 0.01 per replication cycle. This high mutation rate was chosen in order to obtain sufficient levels of genetic diversity. However, we also tested mutation rates up to 10 times lower and found no qualitative differences in the results (data not shown).

In the competition phase the population undergoes recombination in addition to mutation and selection in each reproduction cycle. To this end a recombination modifier locus is added to one end of the genome, with two alleles *m* and *M*, each present in exactly half of the population. Recombination between two genotypes depends on the modifier allele in both genotypes, with the corresponding recombination rates denoted by *r_mm_*, *r_mM_*, and *r_MM_*. For the simulations discussed in the main text we used *r_mm_* = *r_mM_* = 0 and *r_MM_* = 0.1. For this parameter choice individuals carrying distinct modifier alleles cannot exchange genetic material and thus any effect of increased recombination remains linked to the *M* allele. Sexual and asexual individuals compete directly with each other, and we refer to this scenario as the evolution of sex. In contrast, if *r_mm_<r_mM_<r_MM_*, then genetic material can be exchanged between all individuals. We refer to this scenario as the evolution of recombination. For the sake of simplicity, we primarily consider the evolution of sex in the main text, but analogous simulations of the evolution of recombination scenario led to qualitatively indistinguishable results (Text S1). Moreover, for the evolution of sex scenario we also tested values of *r_MM_* ranging from 0.01 to 0.3 (data not shown), which produced qualitatively indistinguishable results. All recombination values refer to a probability of recombination happening between neighboring loci with one recombination event per genome. The position of the crossover point is chosen randomly. No mutations occur between *m* and *M* alleles at the modifier locus.

Recombination, mutation and selection as described above are deterministic and are calculated assuming infinite population size. To examine stochastic effects, we also considered populations with 1,000, 10,000, and 100,000 individuals. Those simulations included a step in which the frequencies of genotypes are sampled from a multinomial distribution according to their frequencies as calculated based on infinite population size.

The burn-in phase always consists of 2500 generations of mutation and selection. We confirmed that 2500 generations were typically sufficient for the system to go into mutation-selection balance from random initial genotype frequencies (data not shown). The competition phase consists of 250 generations of recombination, mutation and selection. For infinite population size we ran a single competition phase for each burn-in phase. For finite-size populations, the outcome was estimated as the average of 100 simulations of the competition phase.

### Predictors for evolution of sex

#### The difference in variance of Hamming distance after full and before recombination, *ΔVar_HD_*

To compute *ΔVar_HD_* we first determine the consensus sequence at the end of the burn-in phase. Next, we compute the variance of the Hamming distances between all sequences and the consensus sequence (i.e. the variance of the number of mutations by which the sequences differ from the consensus sequence). We then compute the variance of Hamming distance after full recombination but before selection. The frequency of any sequence after full recombination is given by the product of the frequencies of its constituent alleles. (When finite population sizes were considered, we create the population after recombination by sampling from the corresponding multinomial distribution.) Full recombination is equivalent to performing recombination with *r* = 1 between all adjacent loci and it completely destroys any linkage disequilibrium. Note that the consensus sequences before and after recombination are identical, because recombination does not change the allele frequencies. Finally, we obtain *ΔVar_HD_* as the difference of the Hamming distance variances in the populations after full recombination and before recombination. Intuitively, *ΔVar_HD_* assesses whether recombination makes a population spread out or become more compact over the space of all possible genotypes. A positive *ΔVar_HD_* may be indicative of an increase in fitness variance and could thus lead to more efficient selection. Mathematically, it can be shown that −*ΔVar_HD_* equals the sum of all pairwise linkage disequilibria with reference to the consensus sequence (Text S1), so we do not consider a separate predictor directly based on linkage disequilibrium measurements. The epistasis-based and the drift-based theories of evolution of sex suggest that negative linkage disequilibrium is a necessary but not sufficient criterion for the evolution of sex [17],[41]. Thus, we expect sex to evolve in our simulation whenever *ΔVar_HD_*>0.

#### The difference of additive genetic variance after full and before recombination, *ΔVar_add_*

We compute the additive genetic variance by fitting log fitness to a linear model that includes only main effects (i.e. the effects of individual loci) but no interactions (i.e. effects that result from combinations of alleles at several loci). We then determine the model parameters *γ_0_* and *γ_j_* such that
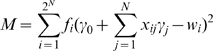
is minimized. Here *f_i_* is the frequency of genotype *i* in the population, *w_i_* is its log fitness, *x_ij_* are binary variables that code for the presence of allele 0 or 1 at locus *j*, *γ_0_* is the estimate of the intercept (i.e. the estimated log fitness of the genotype with allele 0 at all loci), *γ_j_* is the estimated effects of allele 1 at locus *j*, and *N* is the number of loci. The additive genetic variance is then determined as the total variance of log fitness that can be explained by the linear model (i.e. we compute an estimate of the log fitness for all genotypes in the population based on the parameters *γ_0_* and *γ_j_* and then calculate the variance among these estimated fitness values). This procedure is repeated also for the genotypes that are obtained after full recombination. Finally, *ΔVar_add_* is given by the difference in the additive genetic variance in the population after full recombination and at the end of the burn-in phase. Epistasis-based theories suggest that sex is advantageous because it may increase the additive genetic variance and thus lead to more efficient selection, which is frequently referred to as the long-term advantage of sex [28]. Thus, we expect *ΔVar_add_* to be positive in simulations where sex is favored. We also examined total variance in fitness, but results were qualitatively indistinguishable (data not shown).

#### The difference of mean fitness of the population after full and before recombination, *ΔMean_fit_*

We compute *ΔMean_fit_* on the basis of the genotype frequencies at the end of the burn-in phase as the difference in mean fitness after full and before recombination. In populations that are in mutation-selection balance, recombination reduces mean fitness [2] (except in the narrow range for which epistasis measure on a multiplicative and additive scale has different signs). Consequently, we expect *ΔMean_fit_* to be smaller than zero in simulations with infinite population size. However, in simulations with finite population size, due to stochastic effects, *ΔMean_fit_* need not always be negative. Epistasis-based theories have established that the selection on a recombination modifier stems from a long-term and a short-term effect of recombination [28]. As explained above, the long-term effect relates to the effect of recombination on the additive genetic variance. The short-term effect relates to the effect of recombination on mean fitness and is generally detrimental for populations in mutation-selection balance [2],[28]. The theory shows that a recombination modifier can be selected for, provided the beneficial long-term effect outweighs the detrimental short term effect [28]. Thus, in our simulations, we expect selection for sex whenever *ΔMean_fit_* is positive.

#### The strength of physiological epistasis, *E_phys_*

We compute *E_phys_* by first determining the fittest sequence that is present in the population at the end of the burn-in phase. For all possible genotypes (including genotypes that are not present in the population at the end of the burn-in phase) we then measure their Hamming distance to the fittest sequence in the population and regress log fitness *w* against Hamming distance *d* for all genotypes according to *w(n)* = *αn+βn^2^*, where Hamming distance *n* is measured relative to the fittest sequence. The parameter *β* estimates the curvature of the fitness function and is commonly used as a measure of epistasis in experimental work [17], [42]–[48]. Here we use it as the estimate of *E_phys_*. Using the consensus sequence instead of the fittest sequence as the reference did not affect the results qualitatively (data not shown). According to the epitasis-based theories, we expect that in our simulations sex evolves whenever *E_phys_* is negative [2],[28].

#### The strength of population epistasis, *E_pop_*

*E_pop_* differs from *E_phys_* in that this measure is based only on those genotypes that are actually present in the population. Population epistasis is again estimated by regressing log fitness against Hamming distance, but this time only including genotypes that are present in the population, taking into account their relative frequencies. Population epistasis thus quantifies the epistasis that is actually present in population, whereas physiological epistasis measures the epistasis that characterizes the fitness landscape. Epistasis-based theories do not make a clear distinction between population and physiological epistasis, because the assumed parameters (such as the fitness landscape, mutation rate, and population size) fully determine the outcome. However, it is straightforward to construct examples of fitness landscapes in which measures of population and physiological landscapes have opposite sign [17],[19]. Here we expect sex to evolve whenever *E_pop_*<0. As the selection for sex depends on those epistatic interactions that are present in the population we expect *E_pop_* to be a better predictor than *E_phys_*.

#### Predictors based on pairwise epistasis

We measure two predictors that are based on the equations from the Barton's classic paper on evolution of sex [28]. In particular, we consider two ways to combine pairwise measures of epistasis between genes into a single numerical predictor. Based on Equation 12 in [28], if higher-order (than pairwise) interactions are neglected, the change in frequency of a recombination modifier can be approximated according as
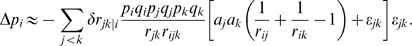
Here, the index *i* denotes the modifier and the sum goes over the selected loci *j* and *k*. The selection coefficients *a_j_* and epistatic interactions 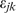 need to be calculated from fitness values and genotype frequencies according to the definitions in [49] and [28]. For the special case of a completely linked modifier (i.e. *r_ij_ = r_ik_ = 0*), on which we focus in our paper, the above expression for the change in modifier frequency is dominated by the long-term effects and becomes proportional to
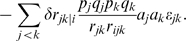
As it is in general quite difficult to obtain experimental data on the location of the modifier and on the rates of recombination between individual loci, we assume that predictors have to be derived in the absence of that knowledge. Hence, the best experimentally feasible predictor that can be derived from the above formula reads
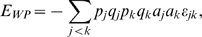
and corresponds to the weighted mean of the epistatic effects. A simplified and frequently used version of this predictor is the (non-weighted) mean of the epistatic effects
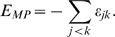
Here we will consider both mean pairwise epistasis (E_MP_) and weighted mean pairwise epistasis (E_WP_) as predictors for the direction of the selection on a modifier of recombination.

## Supporting Information

Text S1 Details of the study's computational aspects; additional experiments for a different modifier value combination; experiments with neutral loci; analytical derivation of the relationship between the variance of Hamming distance and linkage disequilibria in the 2-loci case.
(0.35 MB DOC)
