# Supplementary material for: Predicting the Evolution of Sex on Complex Fitness Landscapes
Source: PLoS Comput Biol. 2009 Sep 18;5(9):e1000510. doi: 10.1371/journal.pcbi.1000510 (PMC2734178; doi:10.1371/journal.pcbi.1000510)
Supplement: Text S1 — Details of the study's computational aspects; additional experiments for a different modifier value combination; experiments with neutral loci; analytical derivation of the relationship between the variance of Hamming distance and linkage disequilibria in the 2-loci case. (0.35 MB DOC) [file pcbi.1000510.s001.doc]

**Supporting Information for**

**Predicting the evolution of sex on complex fitness landscapes**

Dusan Misevic, Roger D. Kouyos, and Sebastian Bonhoeffer

**Computational considerations.** We restricted our simulations to fitness landscapes with 6 loci (or 9 loci when considering the landscapes with neutral loci). The confinement to small landscapes is necessary due to computational constraints. In particular, problems arise in deterministic simulations, where we must keep track of all possible genotypes, even when they are present with infinitesimally small frequencies. Since there are 2*^N^* genotypes for landscapes with *N* loci, this quickly becomes unfeasible for large *N*. In simulations with finite population size the problem does not occur, because the number of genotypes present is typically substantially smaller than the population size. However, for stochastic simulations a different computational limitation arises, as it is necessary to run many instances for a given set of parameters to determine the average outcome. In order to enable a comparison between stochastic simulations with finite population size and deterministic simulations with infinite population size, here we chose to perform simulations using genomes with 6 loci. All programs used for the simulations shown here were developed in the R or C programming language.

**Evolution of recombination.** In the main text, we have focused on the evolution of sex scenario, where the recombination rates for different combinations of modifier alleles were *r_mm_* = *r_mM_* = 0 and *r_MM_* = 0.1. We referred to this parameter setting as the evolution of sex, because the competition occurs between purely sexual and purely asexual individuals. In contrast, when 0 ≤ *r_mm_ < r_mM_ < r_MM_*, individuals carrying distinct modifier alleles can exchange genetic material. We repeated all the simulations from the main text with *r_mm_= 0*, *r_mM_ = 0.05* and *r_MM_ = 0.1,* without changing the other parameters. Effectively, here recombination can occur even if one of the individuals carries allele *m*. Because the focus is on the varying degrees of recombination, we refer to this scenario as the evolution of recombination. We found that the main results for the evolution of recombination were qualitatively indistinguishable from the ones obtained under the evolution of sex settings. In particular, all predictors preformed well on smooth landscapes, but only *ΔVar_HD_* and *E_pop_* had high accuracy across all landscapes tested (Figure S1). Simple combinations of predictors are not likely to significantly increase the overall accuracy of predictors (Figure S2). Finally, genome neutrality had negative effect on *ΔVar_HD_* and *E_pop_* accuracy, while other predictors remained ineffective (data not shown).

**Neutral loci.** In order to address the effect of neutral loci on the predictors described in the main text, for each previously used landscape we generated a corresponding landscape by adding three neutral loci to one end of the genome. These landscapes are thus based on 9 biallelic loci. We ran the burn-in phase 100 times for each of the 810 landscapes (as opposed to only once, as done before). It is not necessary to re-run the competition phase on the new landscapes, since neutral loci do not affect the evolution of sex. Here, we also do not simulate populations of infinite size, because we are interested in the stochastic effects on allele frequencies at neutral loci. The same procedure was repeated for each for the three population sizes (1,000, 10,000, and 100,000), with all other simulation parameters being the same as before. As expected, the predictors *ΔVar_add_* and *ΔMean_fit_* do not change by adding neutral loci (data not shown). However, the other 3 predictors *ΔVar_HD_*, *E_phys_*, and *E_pop_* do change in the landscapes we used.

To assess whether including neutral loci further decreases the accuracy of these predictors we compared the predictors calculated on the basis of all 9 loci (6 selected and 3 neutral loci) to the corresponding predictors based on the 6 selected loci only (by adding up the genotype frequencies over all neutral loci). The panel A of Figure S3 shows the consistency between the predictions based on 6 versus 9 loci, i.e the fraction of cases in which the prediction based on all 9 loci agree with that based on the 6 selected loci for the three chosen population sizes. For *E_phys_* the agreement between the predictions based on 9 and 6 loci is generally high, while *E_MP_* and *E_WP_* are not affected by neutral loci. The agreement between the predictions based on 9 versus 6 loci is much poorer for *ΔVar_HD_* and *E_pop_*. Thus, while these two are typically robust across different types of landscapes (Figure 1, main text), their predictive quality is compromised when neutral loci are included. On the other hand, *E_phys_*, *E_MP_*, and *E_WP_* are robust to including neutral loci, they are generally poor predictors across different types of landscapes.

The panel B of Figure S3 shows that three out of seven predictors considered (*ΔVar_HD_*, *E_phys_*, and *E_pop_*) exhibit a systematic bias when neutral loci are present. The bias in *E_phys_* is due to the fact that “stretching” the decline of the fitness function over 9 rather than 6 loci decreases its curvature (and slope). However, this stretching does not affect the sign of the curvature, explaining why the predictors based on 9 and 6 loci generally agree qualitatively. The estimates of *E_pop_* based on 6 or 9 loci, however, do not need to agree with regard to their sign, because random fluctuation of the neutral alleles can shift the weights in the regression of log fitness against Hamming distance in ways that result in a change of sign for the estimation of *E_pop_*. Such random fluctuations are especially frequent in simulations with small population size, when stochastic effects are strong (e.g. *E_pop_* in simulations with 1,000 individuals, Figure S3, panel B)

*ΔVar_HD_* exhibits a weak systematic bias for the landscapes tested. An intuition for the source of this bias can be obtained by considering the two loci case. For two loci the linkage disequilibrium is defined as *D = f_AB_ f_ab_ – f_aB_ f_Ab_*, where *f_xy_* are the frequencies of the genotypes and *A/a* and *B/b* are the two alleles at the two loci. It is straightforward to verify that *D =* -*ΔVar_HD_* if *AB* is the genotype of the consensus sequence (see section below). If we consider a completely neutral situation (i.e. all genotypes have the same fitness), then all genotype frequencies have a priori the same expectation value. The expected frequency of the consensus sequence, *f_AB_*, however, is larger than the expected frequency of the other genotypes, *f_ab_*, *f_aB_*, and *f_Ab_*. This is the case because for two loci the consensus sequence is defined as that sequence for which the sum of twice its own frequency plus the frequency of its 1-point mutations is maximal and thus genotypes with high frequency have a greater chance of being identical to the consensus sequence. As a consequence, estimating *D* with regard to the consensus sequence results in a bias towards positive values, and thus a bias towards negative values for *ΔVar_HD_*. Hence, the difference between *ΔVar_HD_* calculated based on 6 and 9 loci is positive. This bias is small across all landscapes tested (Figure S3, panel B) but can become large in near neutral landscapes (see Figure S4).

**Variance of Hamming distance and linkage disequilibria.** The following calculation shows that *ΔVar_HD_* equals the sum of pairwise linkage disequilibria with the consensus sequence as a reference. If we denote a genotype by

and the consensus sequence by

,

then the Hamming distance to the consensus sequence can be written as

where the function *y* is defined by

.

If furthermore *f(x)* denotes the frequency of the genotype *x, f(x_i_)* the frequency of allele *x_i_* and *f(x_i_ ,x_j_)* the frequency of the allele combination (*x_i_ , x_j_*), the variance of *h* can be calculated as follows:

with

and

.

Therefore

,

where $D_{c_{i}c_{j}}$denotes the linkage disequilibrium between loci *i* and *j* with reference to the consensus sequence. Thus the variance of the Hamming distance corresponds to the sum of all pairwise linkage disequilibria plus the sum of the variances of the allele frequencies.

If *r_ij_* denotes the recombination rate between loci *i* and *j*, then one round of recombination reduces $D_{c_{i}c_{j}}$ to . As recombination leaves the allele frequencies unchanged, the change in Hamming distance can be written as

.

The full genome shuffling we perform in our simulations corresponds to *r_ij_* = 1 (i.e. the linkage disequilibria are destroyed completely) and thus

.


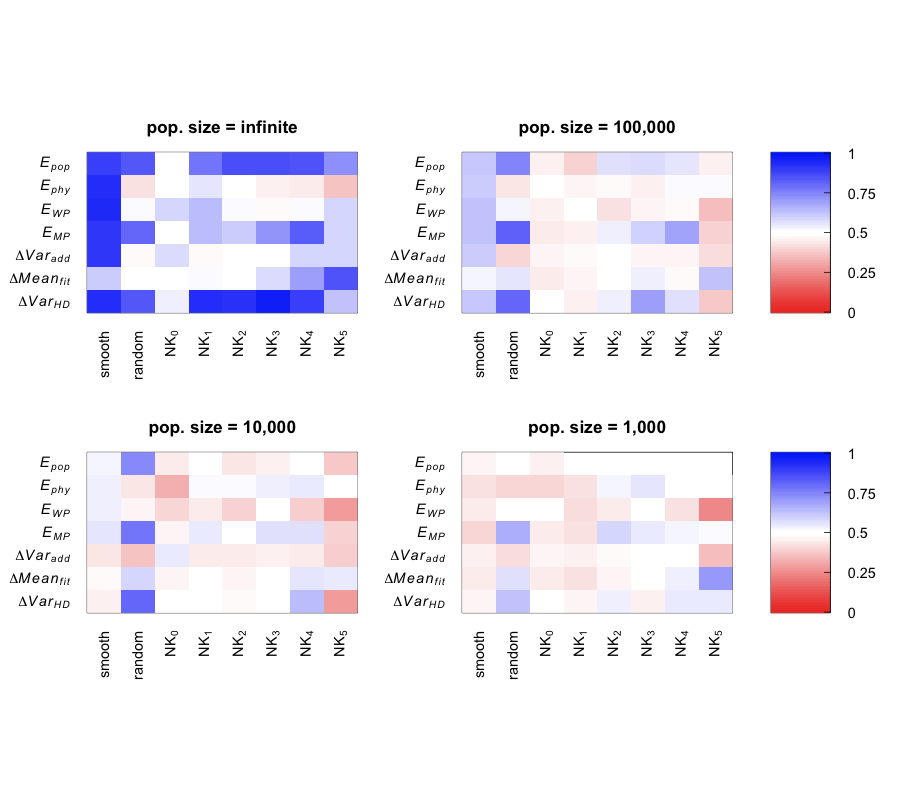


**Figure S1:** Predictor accuracy for different landscape types. Panels correspond to simulations with different population size. Recombination rates used are *r_mm_= 0*, *r_mM_ = 0.05* and *r_MM_ = 0.1* and correspond to the evolution of recombination scenario. Predictors with absolute values smaller than 10^-15^ were considered numerical artifacts and were instead assigned values of -1 or 1 at random. This is appropriate, because all predictors rely on sign and not magnitude in predicting the outcome of the competition phase. Note, that the predictors *E_MP_* and *E_WP_* reflect only the long-term effect of recombination on the selection for a recombination modifier as described in reference [1]. In contrast to the simulations shown in figure 1 in the main text where completely linked modifier is assumed, these two predictors are not perfectly suited from a theoretical point of view for the simulations shown here as they neglect the contribution of the short-term effect. However, from a practical perspective, the contribution of the short-term effect can only be estimated provided there is quantitative information on the linkage between the modifier and the selected loci, which is typically not available for most experimental settings.


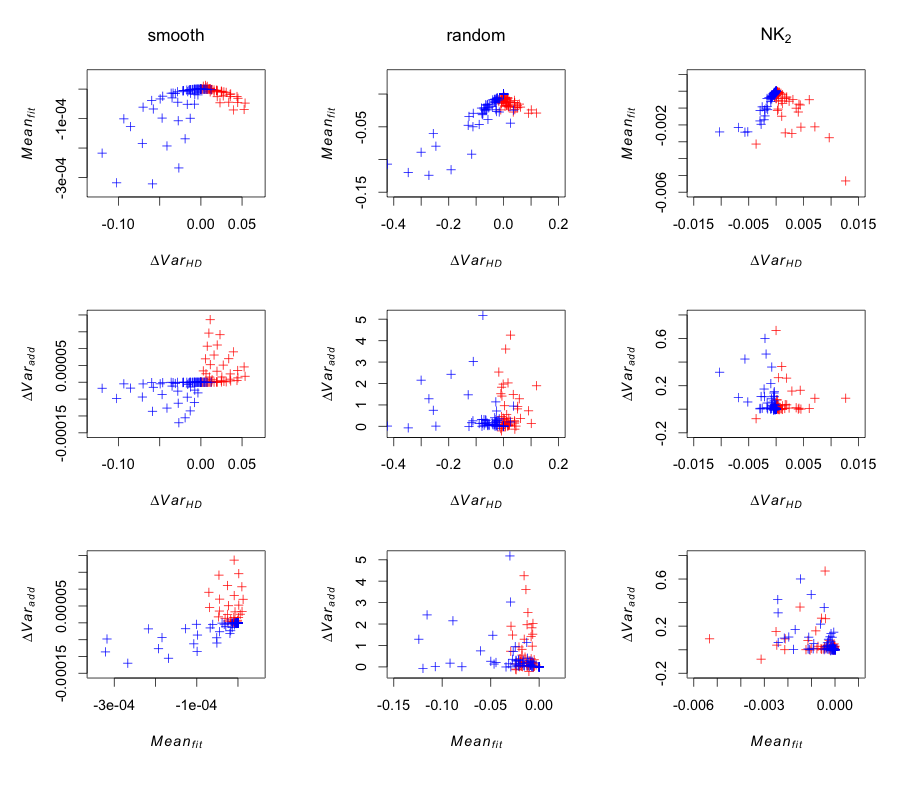


**Figure S2:** Correlations between on different landscape types, recombination rates *r_mm_= 0*, *r_mM_ = 0.05* and *r_MM_ = 0.1,* evolution of recombination scenario. We highlight the relationships among three of the seven predictors (*ΔVar_HD_*, *ΔVar_add_*, and *ΔMean_fit_*) on three of the eight landscapes types (smooth, random, NK_2_). All simulations shown are based on infinite population size. Each cross mark (+) represents a predictor value for a single simulation. Red (blue) crosses indicate simulations in which the frequency of sexually reproducing individuals increased (decreased) in the competition phase. In all cases, simulations were preformed with infinite population size (deterministic case). For clarity of presentation, up to five outlier points were eliminated from random and NK_2_ landscapes. These outliers in predictor values are characteristic of a small number of populations that have not reached the equilibrium genotype frequencies by the end of the burn-in phase.


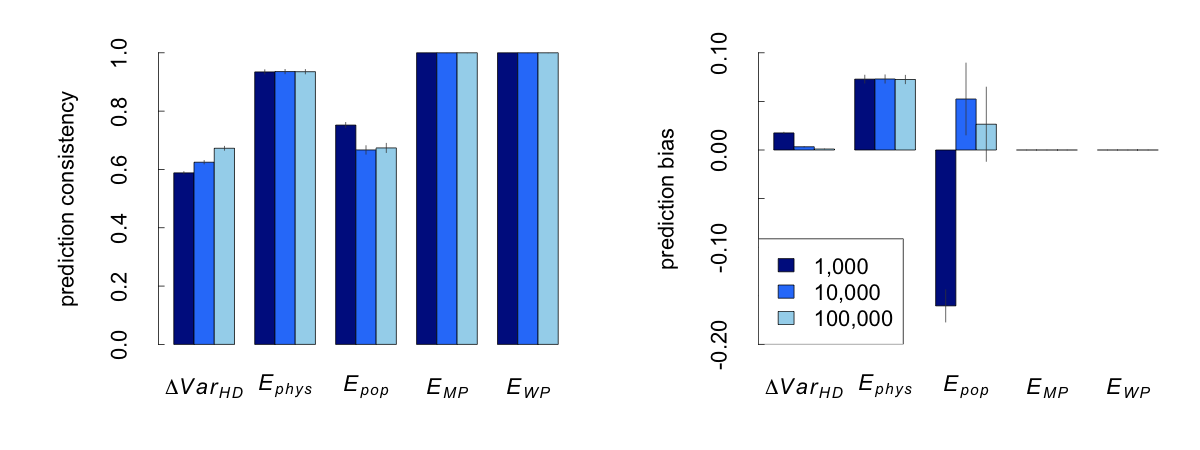


A

B

**Figure S3:** Comparison of predictors calculated based on simulations that used genomes with neutrality (9 loci, 3 of which are neutral) and without neutrality (6 loci). Panel A shows the consistency of the predictions based on the 9 and 6 locus landscapes for the predictors *ΔVar_HD_*, *E_phys_*, *E_pop_*, *E_MP_*, and *E_WP_*. Prediction consistency is measured as the fraction of cases in which predictions about evolution of sex agreed in 6 and 9 loci case. Panel B shows the systematic bias that arises from including neutral loci. The prediction bias is calculated by subtracting the values of the predictors in 9-loci case from the values obtained in the 6-loci case. In both panels, the value of each is the mean of the pooled data from the simulations conducted with population sizes 100,000, 10,000 and 1,000 on all types of landscapes; error bars represent one standard error of the mean.


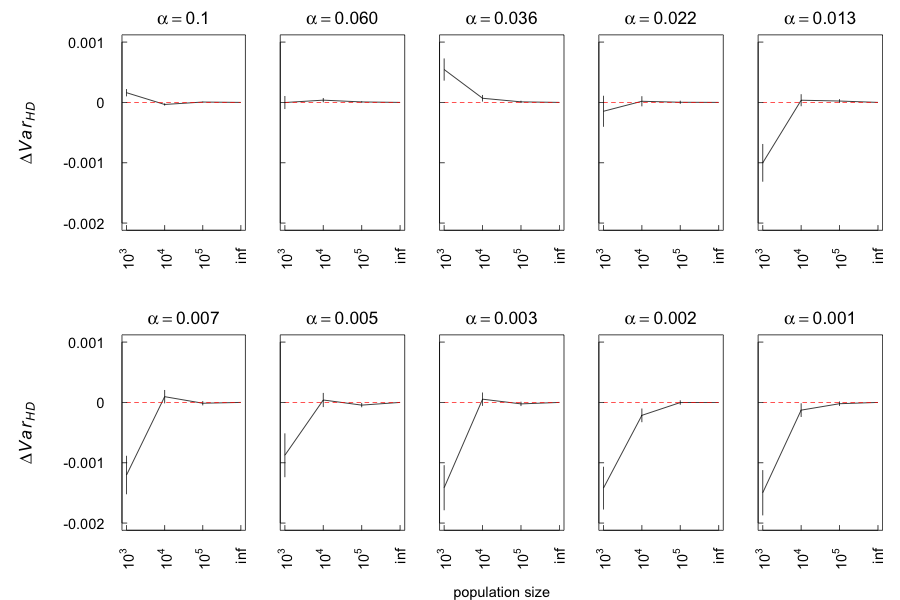


**Figure S4:** Bias in *ΔVar_HD_* on smooth landscapes without epistasis (*β =* 0). For each value of *α*, the mean *ΔVar_HD_* over 1000 simulations is plotted for populations of size l0^3^, 10^4^, 10^5^, and of infinite size. In all plots, dashed line is *ΔVar_HD_* = 0; error bars represent one standard error of the mean. A systematic bias in *ΔVar_HD_* can be observed specifically in simulations with small population sizes and with weak selection coefficients. Under those conditions, the consensus sequence is often not identical to the fittest sequence, resulting in a biased estimation of *ΔVar_HD_*. The positive values of *ΔVar_HD_* for high selection coefficients and small population sizes are likely due to the Hill-Robertson effect that generates negative linkage disequilibria (and thus positive *ΔVar_HD_*) due the combined force of drift and selection.

**References**

1. Barton NH (1995) A general model for the evolution of recombination. Genet Res 65: 123-144.
